# Immune response to hepatitis B vaccine following complete immunization of children attending two regional hospitals in the Southwest region of Cameroon: a cross sectional study

**DOI:** 10.1186/s12879-021-06913-y

**Published:** 2021-12-02

**Authors:** Ephesians N. Anutebeh, Lambed Tatah, Vitalis F. Feteh, Desmond Aroke, Jules C. N. Assob, Simeon Pierre Choukem

**Affiliations:** 1 Faculty of Health Sciences, University of Buea, Buea, Cameroon; 2 Health and Human Development (2HD) Research Network, Douala, Cameroon; 3 MRC Epidemiology Unit, University of Cambridge, Cambridge, UK; 4 Nuffield Department of Medicine, Big Data Institute, University of Oxford, Oxford, UK; 5 Newark Beth Israel Medical Centre, Newark, NJ USA; 6 African Journal of Integrated Health, Buea, Cameroon; 7 Faculty of Medicine and Pharmaceutical Sciences, University of Dschang, Dschang, Cameroon; 8 Faculty of Medicine and Pharmaceutical Sciences, University of Douala, Douala, Cameroon

**Keywords:** Hepatitis B vaccine, Immune Response, Infants, EPI, Cameroon

## Abstract

**Background:**

Hepatitis B virus (HBV) infection despite being a vaccine preventable disease remains a global public health problem. In Cameroon, the hepatitis B vaccine was introduced in the expanded program on immunisation in 2005, but there has been limited evaluation of the HBV surface antibody response post vaccination.

**Objective:**

We investigated the immune response to hepatitis B vaccine in infants who received the DPT-Hep B-Hib vaccine, and we assessed HBsAg carriage in non-responders. We also investigated factors associated with non-response or poor response.

**Methods:**

Using a hospital based cross sectional design and a structured questionnaire over a four-month period (January to April 2019), we collected data to determine factors associated with hepatitis B surface antibody (anti-HBs) response from infants aged 6 to 9 months attending infant welfare clinics (IWC) at the Buea and Limbe regional hospitals. We collected venous blood and measured anti-HBs titres using a quantitative Foresight® ELISA. We entered and analysed data using EpiData version 3.1 and SPSS version 25 respectively.

**Results:**

Of the 161 infants enrolled, 159 (98.8%) developed anti-HBs antibodies. Of these 159, 157 (97.5%) and 117 (72.7%) developed ≥ 10.0 mIU/ml (seroprotection) and ≥ 100.0 mIU/ml anti-HBs titres respectively. Being younger (6 months old) was associated with seroprotection (Cramer V = 0.322, p = 0.001). Spearman rho’s relational analysis showed that immunity against HBV reduced as the duration since the last dose increased (r = −0.172; P = 0.029). However, a Firth logistic regression showed no significant association of factors with inadequate immunity. All 12 (7.5%) infants exposed to HBV at birth, received the hepatitis B vaccine at birth, including four who received HBIG, and all were protected. Four infants (2.5%) had anti-HBs titres < 10.0 mIU/mL (non-responders) but had no peculiarity.

**Conclusion:**

The seroprotective rate following hepatitis B vaccination of infants is high even in exposed infants. Our study suggests that Cameroon’s HBV vaccine in the Expanded Program on Immunisation (EPI) is effective against HBV, although we could not account for the 2.5% non-response rate. Large scale studies are needed to further explore non-response to the vaccine.

## Introduction

Hepatitis B virus (HBV) is a DNA virus of the Hepadnaviridae family, and it is responsible for both acute/chronic liver pathology [1]. About a third of the world’s population have serologic evidence of current or past HBV infection [2]. Of these, an estimated 257 million are chronic carriers and 887,000 persons die annually due to HBV infection or it’s complications [3]. Although HBV has a global spread, the geographical patterns of its prevalence vary hugely. Regions are divided into low, intermediate, and high endemicity. In Sub-Saharan Africa, HBV is highly endemic with a HBsAg prevalence ranging from 5 to 10% [4], and Cameroon stands out with an estimated prevalence of 11.2% [5].

In highly endemic areas, HBV in infants is most spread through perinatal transmission. Infection with HBV can be asymptomatic or can cause acute hepatitis. These conditions either resolve spontaneously with subsequent immunity or lead to a chronic infection that is lifelong [6]. About 90% of infants infected within the first year of life will progress to a chronic disease state. However, the rate of progression to chronic infection decreases with increasing age of primary acquisition [7, 8].

Vaccination of infants and neonates against the HBV infection has been found to greatly decrease the burden of the disease [9]. A safe and effective vaccine has been available since 1982, and only started in Cameroon in 2005. The goal is to prevent HBV infection and its complications [1, 7]. Given the high success with vaccination, the World Health Organisation has recommended the routine screening of pregnant women and implementation of appropriate measures during childbirth to reduce transmission, including the vaccination of every newborn. Testing and vaccination of adults who test negative for HBsAg are also recommended measures to curb the spread of HBV. A course of three doses of hepatitis B vaccine induces protective levels of antibody to HBsAg in over 95% of healthy infants and children [10, 11]. Antibody titres above 10 mIU/ml are protective [12, 13]. However, effectiveness of the vaccine is influenced by host factors (age, comorbidity, prior exposure to HBV, and time since vaccination) and vaccine-related factors (type, dose and schedule of vaccine used) [9, 14, 15]. There has been a decrease in the proportion of chronic HBV infection in African children under 5 years of age from 4.7% in the pre-vaccine era to 1.3% as of 2015 [3].

Generally, about 5–10% of vaccinated infants may not develop the expected immune response following complete HBV vaccination [16]. This is due to several factors ranging from site of vaccination to genetic variability of individuals. Studies have shown that the longer the duration between the second and third doses of the vaccine, the higher the immune response [1].

The Expanded Program on Immunisation (EPI) in Cameroon recommends the vaccination of all newborns, and there are continuous vaccination campaigns to ensure good coverage, with generally high uptake rates (99%) [17]. The EPI uses 6, 10- and 14-weeks schedule with just a month difference between the second and third doses of the vaccine. For exposed newborns, it is recommended that both HBIG and hepatitis B vaccine be administered at birth (seroimmunisation), although HBIG is costly for most mothers. Generally, antibody production post vaccination is 1 month to reach its peak and then gradually starts waning as from 3 months post vaccine. However, no test is done after vaccine completion to identify and measure anti HBs production. This implies that little or no measures are taken to control and confirm the effectiveness of the vaccine or vaccine schedule after administration. In Cameroon, there are few data available on the immune response to hepatitis B vaccine and the vaccine schedule used in the EPI. We aimed to determine the antibody response to hepatitis B vaccine and to evaluate factors associated with no or poor response in two regional hospitals in Cameroon.

## Methods

### Study design, setting, and population

We undertook a cross-sectional study in the Southwest region of Cameroon, where we recruited participants over a period of four months (January to April 2019). Our target population was infants attending Infant Welfare Clinics (IWC) in the Buea Regional Hospital (BRH) and Limbe Regional Hospital (LRH). These hospitals are the major hospitals in the Southwest region of Cameroon, and they each receive averagely 80–100 infants per month for routine vaccination visits. These facilities have well equipped laboratories with the materials needed for sample collection, adequate storage, and analysis. Even though the LRH has multiple IWC visit days per week and a larger general population compared to BRH, we did not intend to compare results between the two hospitals.

### Sampling method

All infants aged 6–9 months with documented evidence of receiving all three doses of the HBV vaccine and last dose received at least a month prior to the study were sampled for this study. Infants were met at the various IWC Centres and the study explained to the parents or guardians. Those who consented to participation orally then signed a written consent and were enrolled. We excluded all infants with documented evidence of HBV infection and further excluded infants with insufficient blood samples.

### Sample size estimation

Our minimum sample size of 114 participants was calculated using the Cochran’s formula [18]:

${\varvec{n}}=\frac{{{\varvec{Z}}}^{2}{\varvec{p}}(1-{\varvec{p}})}{{{\varvec{d}}}^{2}}$ where n = minimum sample size, Z = Standard normal Variation which was set at 1.96 (p value: 0.05 and 95% confidence interval), p = anti-HBs seroprevalence in immunized infants. A study carried out in Dakar (Senegal) and Yaoundé (Cameroon) revealed a prevalence of 92% [19]. Thus, we assumed a p = 92% = 0.92, and d = precision at 0.05 at 95% confidence interval.

#### Study procedure

We approached parents at the IWC, gave talks on general infant welfare and about the HBV. Participants with infants between 6 and 9 months were approached, study explained to them, and consent obtained.

### Laboratory analysis

#### Specimen collection, transportation, and storage

Following participants enrolment, venous blood samples were collected using a 5-ml syringe into a 4-ml serum separating tube. These samples were then transported within 30 min following collection in a blood transport box to the laboratory where samples were centrifuged, serum extracted and stored at −20 °C. ELISA kits were stored at 4 °C.

### Assessment of Hepatitis B surface antibody (anti-HBs) titres

Commercial quantitative ELISA Foresight® (ACON Laboratories, Inc., USA) was used to determine the presence of antibodies to HBsAg. Tests were performed according to the kit manufacturer’s instructions using sandwich ELISA method Foresight® (ACON Laboratories, Inc., USA).

Reagents and samples were removed and allowed to thaw prior to testing. We then prepared the working wash buffer (Tris–HCl). For each micro titre plate used, well A1 was left as blank. Calibrators (Standards obtained from the manufacturer containing varying known concentrations of anti-HBs and negative for HBsAg, HCV, and HIV) preserved in 0.1% ProClin™300 were added into wells B1 to C2. The colour of wells containing calibrator 1–5 gradually changed from yellow to Blue. Starting from D2 we added our samples. We added 50 µL of conjugate containing purified HBsAg bound to peroxidase to each well. We then mixed gently the plate and incubated at 37 °C for 30 min. Each well was then washed 5 times with 350 µL of working wash buffer and dried. To each well, 50 µL of substrate A (citrate phosphate buffer containing hydrogen peroxide) and B (buffer containing tetramethylbenzidine [TMB]) were added and incubated at 37 °C for 15 min. A blue coloration was noticed in positive wells. The reaction was then stopped by adding 50 µl of the stop solution (0.5 M H2SO4) to each well. The positive wells that initially developed blue colour changed to yellow while the negative wells (that were initially colourless) remained colourless. All calibrators, buffer, conjugate, substrates and stop solution were from ACON Laboratories, Inc., USA.

The optical densities of the wells were determined immediately using the spectrophotometer at 450 nm–700 nm. The concentration of the standards and their optical densities were used to plot a standard curve from which corresponding concentration of the optical densities of the samples were extrapolated.

In order to identify those with HBsAg carriage, all children who had not developed anti-HBs were screened for HBsAg using Wondfo rapid test (Guangzhou Wonfo Biotech Inc., China).

### Additional data

#### Data collection

A semi-structured interview form was used to obtained sociodemographic data and medical information to assess variables such as vaccination status of infants [number of doses, interval between doses, route of administration, use of birth dose of vaccine and use of hepatitis B immunoglobulin (HBIG)], vaccination timeliness, mother’s hepatitis B status prior to parturition and immune status. Infants’ medical histories (gestational age, birthweight, and past illnesses) were obtained from past records and current anthropometric parameters were measured and recorded to complete the questionnaire by the investigator.

### Data analysis

Participants were identified using unique codes and data were inputted into a secured computer using participants codes only. These data will be deleted at the end of statistical analysis. Data obtained were entered into EpiData version 3.1 and the database exported to IBM SPSS version 25 software (IBM Corporation, Somers, New York) and analysed. The primary study outcome was infants with anti HBs titres > 10.0 IU/µL (seroprotection). Continues variables such as age, gestational age and birthweight were discretized. Participant characteristics were summarised using proportions, frequencies and percentages as well as mean and standard deviation (SD) or median and 25th–75th percentiles as appropriate. Spearman correlation test was performed on all the variables to find out if there was any significant relationship between any two variables. Differences between means of quantitative data were tested using Students’ t-test while association between the categorical variables was determined using chi squared (χ^2^) or Cramer’s V test. A p value < 0.05 was considered statistically significant. We used a Firth logistic regression model with backward deletion for our multivariate analysis since events in our outcome variable were rare.

## Results

### Characteristics of study population

We enrolled 161 infants in the study. The median and interquartile range (IQR) of age of participants were 7 and 3 months. The modal age was 6 months (49.1%). Females were more represented (57.8%), and the majority of our participants enrolled at LRH (65.2%). Twelve (7.5%) participants were born to HBV carriers and all 12 received the vaccine at birth, although only 4/12 (33.3%) of the children born to HBV carrier mothers received the HBIG at birth. The mean (± standard deviation) gestational age at birth was 39 (± 2) weeks and average birthweight was 3.38 (± 0.59) kg. Tables 1 and 2 summarize the sociodemographic and clinical characteristics of our participants respectively.

**Table 1:** Sociodemographic characteristics of mothers and infants attending Infant Welfare Clinics in Limbe and Buea, Cameroon

Variable	Number of participants, N^a^	Percentages (%)
Sex		
Female	93	57.8
Age		
6 months	79	49.1
7 months	9	5.6
8 months	6	3.7
9 months	67	41.6
Place of sampling		
Limbe	105	65.2
Buea	56	34.8
Informant’s level of education		
Primary	18	11.2
Secondary	95	59.0
University	48	29.8
Religion		
Christian	153	95
Muslim	8	5

^a^N, total number of participants (161)

**Table 2:** Clinical Characteristics of infants attending Infant Welfare Clinics in Limbe and Buea, Cameroon

Variable	Number of participants (total)	Percentages (%)
Mother a chronic HBV^a^ carrier	12 (161)	7.5
Baby received HBV^a^ vaccine at birth	12 (12)	100
Received HBIG^b^ at birth	4 (12)	33.3
Mother is HIV^c^ positive	6 (161)	3.7
Gestational Age at Birth		
Preterm	12 (161)	7.5
Term	149 (161)	92.5
Birthweight		
Low birthweight	8 (161)	5
Normal birthweight	138 (161)	85.7
Macrosomia	15 (161)	9.3
Documented past illnesses		
Neonatal infection	18 (161)	11.2
Malaria	33 (161)	20.5
URTI^d^	12 (161)	7.5
Febrile gastroenteritis	4 (161)	2.5
Others^e^	5 (161)	3.1
Nutritional Status		
Weight for age assessment:		
Adequate	158 (161)	98.1
Underweight	2 (161)	1.2
Overweight	1 (161)	0.7

^a^HBV Hepatitis B virus. ^b^HBIG Hepatitis B Immunoglobulin. ^c^HIV Human immunodeficiency virus. ^d^URTI Upper respiratory tract infections. ^e^Others (bronchopneumonia, sepsis, urinary tract infections)

### Vaccination timeliness

The interval between the first and the second dose of hepatitis B vaccine ranged from 1 to 3 months, with the most frequent interval being 1 month (93.2%). The interval between the second and third dose also ranged from 1 to 3 months, with the most frequent interval being 1 month (88.8%). Most of our participants strictly followed the EPI schedule with a 1 month interval between each of the 3 doses (81.4%: 95% CI 74.8–86.8). The duration since last dose of vaccine ranged from 1 to 7 months with a median and IQR of 4 and 3 months respectively, and most represented being 3 months (24.8%) prior to study. Table 3 summarises vaccination timeliness of participants.

**Table 3:** Different vaccine schedules followed by infants in Cameroon

Variable	Number of participants (n = 161)	Percentages (%)
Interval between 1st and 2nd dose of HBV^a^ vaccine		
1 month	150	93.2
2 months	9	5.6
3 months	2	1.2
Interval between 2nd and 3rd dose of HBV^a^ vaccine		
1 month	143	88.8
2 months	17	10.6
3 months	1	0.6
Received HBV^a^ vaccine on scheduled time according to EPI^b^		
Yes	131	81.4
No	30	18.6
Duration since last dose of HBV^a^ vaccine		
2 months	39	24.2
3 months	40	24.8
4 months	18	11.2
5 months	31	19.3
6 months	29	18.0
7 months	4	2.5

^a^HBV Hepatitis B virus. ^b^EPI Expanded program on immunisation (Interval between vaccine doses 1 month apart)

### Prevalence of adequate immunization

The anti-HBs antibody titre of participants ranged from 0.0 to 257.7 mIU/ml with a median (25th, 75th percentile) titre of 197.8 (95.9, 205.6) mIU/ml. Most participants 117 (72.7%) had a high response. Participants with immune response > 10.0 mIU/ml were considered protected against the HBV infection. Of the 161 participants, 157 had anti-HBs titres > 10.0 mIU/ml giving a prevalence of 97.5% (95% CI 94.1–99.2).

### Factors associated with adequate immune response

Younger age was found to be significantly associated with high levels of anti-HBs. Also, in our study, a relational analysis between duration since last dose of vaccine and anti-HBs titre showed a negative correlation, meaning antibody titres had decreased as duration increased. There was no association between anti-HBs response and timely receipt of vaccine. Table 4 summarises factors that might have influenced the immune response. A Firth logistic regression showed no significant association of exposures with outcome variables; however, the events were very rare (2/161).

**Table 4:** Factors associated with Immune response

Factor	All participants N^a^ (%)	Adequate immune response n^b^ (%)	P value
Male	68 (42.2)	66 (97.1)	0.750
Younger age (6 months)	79 (49.1)	79 (100)	**0.001**
Mother attaining secondary Education	95 (59)	94 (98.9)	0.356
Born to Chronic HBV^c^ carrier	12 (7.5)	12 (100)	0.548
Received HBV^c^ vaccine at Birth	12 (7.5)	12 (100)	0.548
Received HBIG^d^ at birth	4 (2.5)	4 (100)	0.438
Born at term	149 (92.5)	146 (98)	0.643
Normal Birthweight	138 (85.7)	134 (97.1)	0.710
Neonatal Infection	18 (11.2)	18 (100)	0.472
Documented febrile illnesses	47 (29.2)	47 (100)	0.193
Mother HIV positive	6 (3.7)	6 (100)	0.690
Adequate weight for height for age	139 (86.3)	135 (97.1)	0.723
Duration since last vaccine dose > 3 months	82 (50.9)	80 (97.4)	**0.029**^**f**^
Follows EPI^e^ strictly	131 (81.4)	128 (97.7)	0.741

Bold values represent statistically significant association of variable with anti-HBs

^a^N total number of infants in a particular group. ^b^n number of infants with adequate immune response within a particular group. ^c^HBV hepatitis b virus. ^d^HBIG hepatitis b immunoglobulin. ^e^EPI expanded program on immunization. ^f^Relational analysis between duration since last dosage and anti HBs titre: Spearman’s Rho *(R = −0.172; P = 0.029)*

## Discussion

In this study we have shown that almost all of our participants including exposed infants, have adequate immune response to HBV following 3 doses of vaccination received during the routine EPI. The observation of adequate immune response did not vary by timing of vaccination and non-responders had no peculiar finding. Younger age and duration since last vaccine dose were significantly associated with anti-HBs levels.

We found that most of our participants (97.5%) mounted adequate immune response following complete vaccination. This finding was in line with the expected global levels of > 95% [3]. However, a previous study done earlier in Yaoundé Cameroon showed a slightly lower prevalence of 92% [19]. This observed minor disparity could be explained by the differences in our study population. The earlier study recruited hospitalised patients up to 4 years of age; sick children will have a lower immune response compared to the healthy population as in our study. Moreover, some previous studies from other sub-Saharan African countries such as Senegal, Ethiopia, Ghana and Nigeria, showed lower seroprotection rates of 58%, 54%, 80.2%, and 61% respectively [19–22]. The different seroprotection rates could be explained by a number of factors some of which include the different genetic makeup of the individuals involved, the different age groups in the various studies and methodological differences including varied biological assays to measure antibody titres. In addition, the effect of the type of vaccine, dosage of vaccine and storage conditions of the vaccine used could not be ruled out as the above variables were not exploited in other studies. It could therefore be ascertaining that, the hepatitis B vaccine schedule used in the EPI in our setting is highly effective in the prevention of the HBV infection.

The non-response rate which stands at 2.5% can be compared to those of other developing and developed countries [23–26]. However, the non-response rate in the current study is lower than the anticipated 5–10% following hepatitis B vaccine immunization in the general population [16, 27]. More so, two out of four of our non-responders did not seroconvert i.e. had no antibodies detected in their serum, they were both females and had no other peculiarities.

Low birthweight, concurrent chronic disease and immunodepression are the most common reasons for non-attainment of seroprotection amongst vaccinated infants. The low prevalence of these factors in our study population could partly explain the excellent levels of seroprotection observed [28]. Genetic influence and measurement errors could be proposed as probable reason for the non-immune response to HBV immunization among our participants [14]. However, more research is therefore warranted to draw meaningful conclusions.

The mean anti-HBs titre of our participants was protective at 153.5 mIU/ml. There was no significant difference in the mean anti-HBs titres of the males (153.8 mIU/ml) compared to the mean anti-HBs titre of females (153.4 mIU/ml) (p = 0.750), which contrast other studies where females responded better to the vaccine than males [29, 30] and/or vice versa [21, 31, 32].Younger age was found to be significantly associated with high levels of anti-HBs. Thus, the older children form the majority of those who do not have protective antibody level, and this is consistent with some studies done in sub-Saharan Africa [20, 21] and Europe [33]. More so, in our study, a relational analysis between duration since last dose of vaccine and anti-HBs titre showed a negative correlation, meaning antibody titres had decreased as duration increased. This finding was similar to that of other studies in sub-Saharan Africa [34, 35]. Thus, as children aged the antibody levels produced following vaccination waned.

In our study, three quarters of our participants received the hepatitis vaccines according to the recommended schedule. This timeliness in vaccine administration can also explain the high rate of vaccine response. However, there was no association between anti-HBs response and timely receipt of vaccine. Equally, there was no association between anti-HBs response and those that did not receive the vaccine according to the exact scheduled times. This observation might suggest that children who have defaulted any given dose of the hepatitis vaccine may be encouraged to resume the schedule on the next available opportunity. However, more data are required to clearly elucidate this.

Overall, most of our participants mounted protective levels of anti-HBs titres and followed strictly the EPI schedule. Increasing age and duration since last dose of the vaccine was found to be associated with declining antibody titre levels. The rest of the characteristics of our participants were not significantly associated with the anti-HBs titre levels. No peculiarity of investigated risk factors associated with non-response was also noticed in the non-responder population, thus opening windows for questioning of an understudied genetic factor.

## Strengths and limitations

Our study is one of the few studies on immune response to the HBV vaccine in a resource limited setting, Cameroon. Also, our study is the first in a semi urban setting, amongst healthy children and we reached the estimated sample size.

Despite the above findings, our study was limited by its cross-sectional design, thus providing just a onetime snapshot of the immune status of our respondents post vaccination. In addition, our questionnaire required informants to recall information, thus allowing room for recall bias. Also, our data are limited to questions asked in the questionnaire, thus, there are factors that are likely to have an influence on immune response that we could not account for from our collected data.

## Conclusion

We found a high seroprotective rate amongst vaccinated infants in our study; therefore, parents and caregivers should continuously be encouraged to vaccinate their children. Children under one year with a missed dose of HBV should resume the vaccination at the next available opportunity, and vaccine providers should follow the national guidelines to increase vaccine access to all infants. The age and time elapse since last dose of the vaccine inversely correlates with the anti-HBs response. Thus, it is important to establish a nation-wide monitoring process to determine immunity post vaccination. We recommend the need for similar studies to cover larger parts of the country, longitudinal studies to assess the long-term immune response following complete immunisation and further studies to evaluate for HBsAg positivity (i.e., to detect the incidence of HBV infection in infants), given the success of vaccination.

## Data Availability

The datasets used and/or analysed during the current study are available from the corresponding author on reasonable request.

## Abbreviations

Anti-HBs: Antibody to hepatitis B surface antigen
BRH: Buea Regional Hospital.
CI: Confidence interval (95%)
DPT-HepB-Hib: Diphtheria Pertussis Tetanus- Haemophilus influenzae b– hepatitis B
ELISA: Enzyme linked immunosorbent assay.
EPI: Expanded Programme on Immunization
FHS: Faculty of health sciences
HBIG: Hepatitis B immunoglobulins (passive immunization)
HBsAg: Hepatitis B surface antigen
HBV: Hepatitis B virus
HCC: Hepatocellular carcinoma
HIV: Human immunodeficiency virus
IWC: Infant Welfare Clinic
LRH: Limbe Regional Hospital
UB: University of Buea
WHO: World Health Organization
